# A Prospective Phase II Study of Pegaspargase-COEP Plus Radiotherapy in Patients With Newly Diagnosed Extra-Nodal NK/T-Cell Lymphoma

**DOI:** 10.3389/fonc.2022.839252

**Published:** 2022-02-25

**Authors:** Shaoxuan Hu, Ningjing Lin, Jiaxin Liu, Yan Sun, Weiping Liu, Xiaopei Wang, Yan Xie, Yuqin Song, Yi Wen, Jun Zhu

**Affiliations:** ^1^ Key Laboratory of Carcinogenesis and Translational Research (Ministry of Education), Department of Lymphoma, Peking University Cancer Hospital & Institute, Beijing, China; ^2^ Key Laboratory of Carcinogenesis and Translational Research (Ministry of Education), Department of Radiation Oncology, Peking University Cancer Hospital & Institute, Beijing, China; ^3^ Medical Department, Medpison (Beijing) Medical Technology Co., Ltd., Beijing, China

**Keywords:** Nk/T cell lymphma, chemotherapy, radiotherapy, ENKTL, treatment

## Abstract

**Background:**

The optimal first-line treatment for extra-nodal NK/T-cell lymphoma (ENKTL) has not been well-defined. This study aimed to evaluate the efficacy and safety of pegaspargase, cyclophosphamide, vincristine, etoposide and prednisone (COEPL) regimen combined with radiotherapy for patients with newly diagnosed ENKTL.

**Methods:**

Our study is a prospective, open-label clinical trial. Patients with newly diagnosed ENKTL and an ECOG performance status of 0 to 2 were eligible for enrollment. For patients with stage I/II nasal ENKTL, treatment included 2 cycles of induction COEPL regimen followed by concurrent chemoradiotherapy, then by 2 cycles of COEPL regimen as consolidation. For patients with stage III/IV or primary extra-nasal ENKTL, treatment included 6-8 cycles of COEPL regimen with or without radiotherapy to local sites, and autologous stem cell transplantation was given in selected patients.

**Results:**

A total of 80 patients were enrolled. The median age was 41 years (range, 15-76 years). Sixteen patients (20%) had stage III/IV disease, and 10 (12.5%) had a PINK score≥2. Complete response and overall response rates were 75.9% and 87.3%, respectively. With a median follow-up of 41.4 months (range 2.7-76.2 months), the 3-year progression-free survival (PFS) and overall survival (OS) rates were 71.3% (95%CI 61.1-81.5%) and 73.3% (95%CI 63.1-83.5%), respectively. For patients with stage I/II nasal ENKTL (n=62), the 3-year PFS and OS were 78.1% and 81.2%, respectively. For patients with stage III/IV or primary extra-nasal ENKTL (n=18), 3-year PFS and OS were 48.1% and 45.7%, respectively. Major grade 3-4 adverse events were anemia (21.3%), leucopenia (22.5%), neutropenia (18.8%), and thrombocytopenia (7.6%). No treatment-related death was observed.

**Conclusions:**

Pegaspargase-COEP chemotherapy in combination with radiotherapy is highly effective and safe for patients with newly diagnosed ENKTL.

## Background

Extra-nodal NK/T cell lymphoma (ENKTL) is a rare subtype of T-cell malignancy which has a higher incidence in Asia than in Western countries (1, 2). The majority of patients with ENKTL present with localized disease characterized by involvement of the nasal cavity and paranasal area, yet distant extra-nasal involvement can be present in 20-30% of patients at diagnosis (3).

Due to the rarity of this disease, the standard of care for ENKTL has not been well-established. Radiotherapy plays a pivotal role in the treatment of patients with early-stage ENKTL (4). However, except for a small proportion of patients with favorable risk features, radiotherapy alone was associated with a relapse rate of 40-50% (4, 5). Thus, chemotherapy has become part of combined modality therapy for patients with early-stage ENKTL and the mainstay of treatment for patients with advanced-stage disease (6).

ENKTL is associated with a high expression of P-glycoprotein leading to resistance to anthracycline-based chemotherapy regimens (7). Consequently, non-anthracycline- containing chemotherapy are currently recommended for the treatment of ENKTL (6). Nevertheless, due to lack of randomized controlled trials, the optimal non-anthracyline-based regimen for ENKTL has not been well-defined. Asparaginase-based or pegarspargase-based regimens have been reported to improve response rates in patients with ENKTL. One of the widely used regimens, the SMILE regimen, produced high response rate (ORR 79%) and favorable 1-year OS (55%) in patients with advanced-stage ENKTL, yet its toxicity profile raises concern (8). Frequency of grade 3-4 infectious events was up to 61% with SMILE regimen (8), which may interfere with treatment compliance and increase risks of treatment-related mortality. These data highlight the need for exploration of less toxic chemotherapy regimens without compromise of treatment efficacy.

Our previous research found that addition of asparaginase or pegarspargase to CHOP regimen could improve treatment outcome of ENKTL with acceptable toxicity (9, 10). Based on our previous experience and recent understanding of management of ENKTL, we designed a novel combination regimen consisting of cyclophosphamide, etoposide, vincristine, prednisone and pegaspargase (COEPL regimen). The purpose of this study was to evaluate the efficacy and safety of this chemotherapy regimen in a prospective phase 2 trial.

## Methods

### Eligibility Criteria

This study is an open-label, prospective, phase 2, single-center clinical trial. Patients were enrolled between October 20th, 2011 and August 13th, 2016. The eligibility criteria were as follows: patients with confirmed pathological diagnosis of ENKTL as defined by WHO criteria, age ≥14 years, no prior chemotherapy or radiotherapy, Eastern Cooperative Oncology Group (ECOG) performance status of 0-2, at least one measurable lesion, adequate bone marrow function (i.e. hemoglobin ≥80 g/l, absolute neutrophil count ≥ 1.0 × 10^9^/L, platelets ≥ 100 ×10^9^/L), adequate renal function (i.e. serum creatinine ≤177 μmol/L), adequate hepatic function (e.g. total bilirubin ≤ two times the upper limit of normal, and ALT/AST ≤2.5 times the upper limit of normal), and expected survival of more than three months. Exclusion criteria included invasion of lymphoma to central nervous system, pre-existing coagulation disorder, other concomitant neoplasms, severe infection, positive HIV antibody, HBV DNA titer higher than 10^4^ copies/ml in HBsAg-positive patients post antiviral therapy; pregnant or lactating women; women of childbearing age unwilling to take contraceptive measures during the study period. All patients gave their written informed consent before entering the study. This study has been approved by the independent ethics committee of our institution.

### Treatment

The COEPL regimen was repeated every 3 weeks and administrated as follows: cyclophosphamide 750 mg/m^2^ intravenously on day 1, vincristine 1.4 mg/m^2^ (maximum dose 2mg) intravenously on day1, etoposide 60mg/m^2^ intravenously on days 1-3; pegaspargase 2500IU/m^2^ intramuscularly on day 2, prednisone 100mg orally on days 1-5. Premedication was given to patients prior to the administration of pegaspargase to reduce the risk of allergic reaction, including dexamethasone and H-1 antihistamines.

For patients with stage I/II nasal ENKTL, treatment protocol included 2 cycles of induction COEPL chemotherapy followed by concurrent chemoradiotherapy (IMRT with prescribed dose ≥45Gy and 2 cycles of concurrent chemotherapy with pegaspargase, vincristine and prednisone), then by 2 cycles of COEPL regimen as consolidation. The concurrent chemotherapy regimen was administrated as follows: pegaspargase 2500IU/m^2^ intramuscularly on day 1, vincristine 1.4 mg/m^2^ intravenously on day1, prednisone 100mg orally on days 1-5; repeated every 14-21 days.

For patients with stage III/IV or primary extra-nasal ENKTL, treatment protocol consisted of 6-8 cycles of COEPL regimen. In addition, local radiotherapy to residual disease and/or autologous stem cell transplantation (ASCT) was applied in selected patients at the discretion of the treating physicians.

### Staging

The pretreatment evaluation and staging procedures included history taking, physical examination, complete hematological and biochemical tests, nasal endoscopy, bone marrow aspiration and biopsy, magnetic resonance imaging (MRI) of the nasal cavity, and computed tomography (CT) scans of neck, chest, abdomen and pelvis. Whole-body positron emission tomography (PET)/CT scan and Epstein-Barr virus DNA (EBV-DNA) testing were recommended but not mandatory. Clinical staging was performed according to the Ann-Arbor classification system.

### Assessment

The primary endpoint was complete response (CR) rate. Secondary endpoints were overall response rate (ORR), overall survival (OS), progression-free survival (PFS) and toxicity. Tumor responses were assessed every two cycles of chemotherapy and were classified as complete response (CR), partial response (PR), stable disease (SD), or progressive disease (PD) according to the Revised Response Criteria for Lymphoma (11). The final assessment was made one month after the end of treatment. Nasal endoscopy, MRI of the nasal cavity, CT scans, or PET-CT when possible, were used to assess treatment responses and to monitor relapses. Radiologic assessment was performed by the investigators. All adverse reactions were graded each cycle according to the National Cancer Institute Common Toxicity Criteria, version 5.

### Statistical Analysis

OS was defined as the time from diagnosis to death from any cause or the date of last follow-up. PFS was measured from diagnosis to first progression, relapse after response, or death from any cause, or the date of last follow-up. OS and PFS were estimated using the Kaplan-Meier method. Log-rank tests and Cox regression methods were used to analyse prognostic factors for PFS and OS. The impact of different factors on treatment response was evaluated by chi-square test. P value less than 0.05 was considered statistically significant. Data were analyzed using SPSS Statistics 19.0 software.

## Results

### Patient Characteristics

A total of 80 patients were enrolled in this study. The baseline characteristics of the patients were detailed in **Table 1**. The median age was 41 years (range, 15-76 years), with a male predominance (71.3%). The majority (80%) of patients presented with stage I/II disease, and 16 patients (20%) had stage III/IV disease at diagnosis. Six patients (8%) presented with primary extra-nasal ENKTL. The Prognostic Index of Natural Killer Lymphoma (PINK) was categorized as low-risk (0 points) in 50 patients (62.5%), intermediate-risk (1 points) in 20 patients (25%) and high-risk (≥2 points) in 10 patients (12.5%). Among the 43 patients in whom baseline EBV-DNA testing was available, EBV-DNA was elevated in 49% of patients.

**Table 1:** Clinical characteristics of patients with ENKTL.

Parameters	No. (%)
Age (years)	
≤60	67 (83.8)
>60	13 (16.3)
Sex	
Male	57 (71.3)
Female	23 (28.8)
ECOG-PS	
0	41 (51.3)
1	38 (47.5)
2	1 (1.3)
B symptoms	
Yes	35 (43.8)
No	45 (56.3)
Ann-Arbor Stage	
I/II	64 (80.0)
III/IV	16 (20.0)
Primary site	
Nasal	74 (92.5)
Extra-nasal	6 (7.5)
LDH	
Normal	58 (72.5)
Elevated	22 (27.5)
EBV-DNA	
Normal	22 (27.5)
Elevated	21 (26.3)
Not Available	37 (46.3)
PINK	
0	50 (62.5)
1	20 (25.0)
≥2	10 (12.5)

### Treatment and Responses

All patients received the COEPL chemotherapy, with a total of 429 cycles of treatment. The median cycles of chemotherapy in this study was 6 (range 1-8). Sixty-four patients (80%) received local radiotherapy with a median radiation dose of 54Gy (45-70Gy). All patients with stage I/II nasal disease without disease progression after induction chemotherapy received radiotherapy, whereas six patients with stage III/IV or primary extra-nasal disease received radiotherapy. Six (7.5%) patients who achieved complete remission (CR) to first-line therapy received autologous stem cell transplantation as consolidation.

One patient withdrew from this study after 2 cycles of chemotherapy due to personal reasons and were not evaluated for response. Thus, treatment response was assessed in the remaining 79 patients who received the scheduled treatment. The overall response rate (ORR) was 87.3% (69/79). Sixty patients (75.9%) achieved CR, 9 patients (11.4%) achieved partial remission (PR), and 10 patients (12.7%) had progressive disease. The ORR and CR rates were 93.4% (57/61) and 82.0% (50/61) for patients with stage I/II nasal disease, and 66.7% (12/18) and 55.6% (10/18) for patients with stage III/IV or primary extra-nasal disease, respectively. Analyses of prognostic factors showed that CR was significantly associated with stage (P=0.017) and PINK (P=0.02) (**Table 2**).

**Table 2:** Impact of clinical characteristics on treatment response in patients with ENKTL.

Characteristics	CR (%)	Non-CR (%)	P value
Age			
≤60	53 (79.1)	14 (20.9)	0.237
>60	7 (58.3)	5 (41.6)	
Gender			
Male	40 (71.4)	16 (28.6)	0.142
Female	20 (87.0)	3 (13.0)	
ECOG-PS			
0	34 (82.9)	7 (17.1)	0.217
1	25 (67.6)	12 (32.4)	
2	1 (100.0)	0 (0.0)	
B symptoms			
Absent	36 (81.8)	8 (18.2)	0.171
Present	24 (68.6)	11 (31.4)	
Ann-Arbor Stage			
I/II	52 (82.5)	11 (17.5)	**0.017**
III/IV	8 (50.0)	8 (50.0)	
Primary site			
nasal	56 (76.7)	17 (23.3)	0.955
Extra-nasal	4 (66.7)	2 (33.3)	
LDH			
Normal	46 (80.7)	11 (19.3)	0.112
Elevated	14 (63.6)	8 (36.4)	
PINK			
0	43 (86.0)	7 (14.0)	**0.020**
1	12 (63.2)	7 (36.8)	
≥2	5 (50.0)	5 (50.0)	

bold values means statistically significant P value, i.e. P value < 0.05.

### Survival

**Figure 1:**
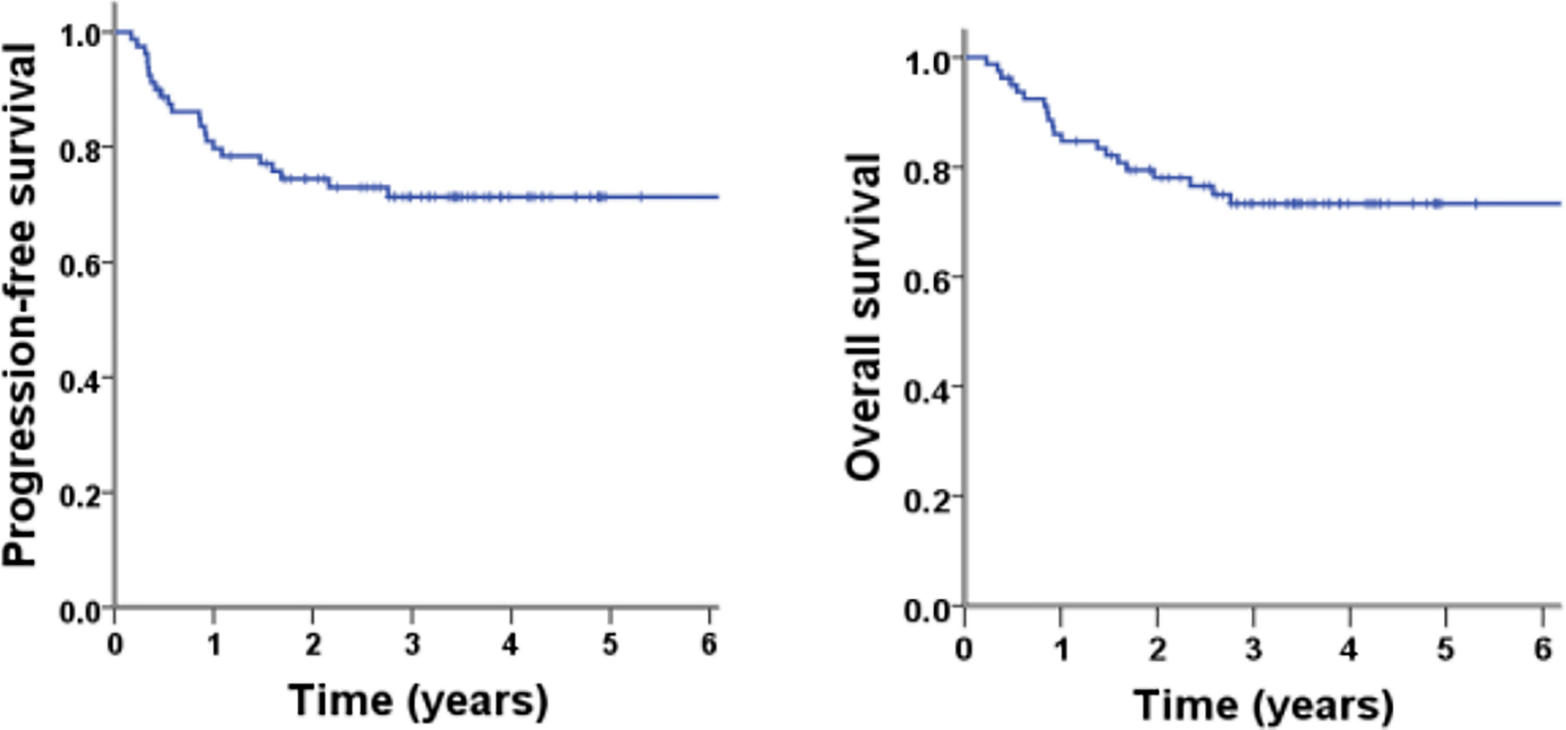
PFS and OS of the patients with ENKTL in this study (N = 80).

**Figure 2:**
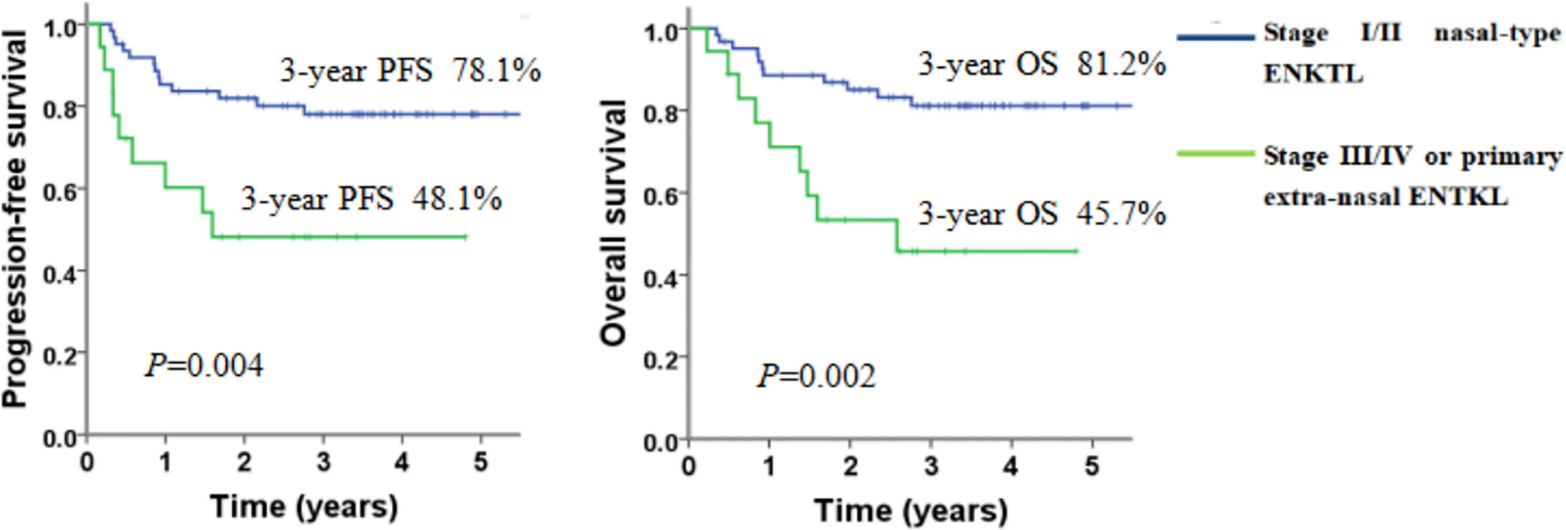
PFS and OS of patients with ENKTL according to clinical stages at diagnosis.

All of the 80 patients were eligible for survival analyses. The median follow-up time for all the patients was 41.4 months (range, 2.7-76.2 months). The median PFS and OS have not been reached. The 3-year PFS and OS rates were 71.3% (95%CI 61.1-81.5%) and 73.3% (95%CI 63.1-83.5%), respectively (**Figure 1**). For the patients with stage I/II nasal ENKTL (n=62), the 3-year PFS and OS were 78.1% and 81.2%, respectively. For the patients with stage III/IV or primary extra-nasal ENKTL (n=18), 3-year PFS and OS were 48.1% and 45.7%, respectively (**Figure 2**). Specifically, of the 6 patients with primary extra-nasal ENKTL, 4 patients remained disease-free after follow-up duration of 20-38 months, and the other 2 patients developed progressive disease and died after 7.4 and 10 months from diagnosis, respectively.

Of the 6 patients with intermediate- or high-risk PINK score who underwent first-line autologous stem cell transplantation, 5 remain disease-free at follow-up duration of 20-57 months, and the other patient relapsed and died 16.5 months after initial diagnosis.

### Prognostic Factors

Results of prognostic analyses for PFS and OS were shown in **Table 3**. In univariate analyses, age, stage and PINK were significantly associated with both PFS and OS. In multivariate analyses, PINK was the only significant factor associated with PFS (P<0.001) and OS (P<0.001).

**Table 3:** Prognostic analyses for PFS and OS in patients with ENKTL.

Parameters	P value		Hazard ratio	95%CI
	Univariate analyses	Multivariate analyses		
PFS				
Age >60 years	0.001	–	–	–
Stage III/IV	0.001	–		
PINK	<0.001	<0.001	3.23	2.09-4.99
OS				
Age >60 years	<0.001	–	–	–
Stage III/IV	0.001	–	–	–
PINK	<0.001	<0.001	2.95	1.97-4.43

### Toxicity Profiles

Adverse events in this study were mainly hematological (See **Table 4**). Grade 3 or 4 anemia, leucopenia, neutropenia, and thrombocytopenia occurred in 21.3%, 22.5%, 18.8% and 7.6% of all patients, respectively. Four patients (5%) experienced febrile neutropenia during treatment. The most common non-hematological toxicity were liver dysfunction and nausea/vomiting, and most were grade 1 or 2. No treatment-related death was observed in our study.

**Table 4:** Adverse Events of the patients with ENKTL in this study.

Adverse Events	No. (%)					
	All	Grade 1	Grade 2	Grade 3	Grade 4	Grade 5
Anemia	66 (82.5)	16 (20.0)	33 (41.3)	17 (21.3)	0 (0.0)	0 (0.0)
Leucocytopenia	56 (70.0)	22 (27.5)	16 (20.0)	8 (10.0)	10 (12.5)	0 (0.0)
Neutropenia	43 (53.8)	11 (13.8)	17 (21.3)	3 (3.8)	12 (15.0)	0 (0.0)
Thrombocytopenia	11 (16.3)	4 (5.0)	3 (3.8)	3 (3.8)	3 (3.8)	0 (0.0)
Nausea	37 (46.3)	22 (27.5)	11 (13.8)	4 (5.0)	0 (0.0)	0 (0.0)
Vomitting	22 (27.5)	12 (15.0)	7 (8.8)	3 (3.8)	0 (0.0)	0 (0.0)
Diarrhea	3 (3.8)	3 (3.8)	0 (0.0)	0 (0.0)	0 (0.0)	0 (0.0)
ALT elevation	37 (46.3)	29 (36.3)	5 (6.3)	3 (3.8)	0 (0.0)	0 (0.0)
AST elevation	37 (46.3)	36 (45.0)	1 (1.3)	0 (0.0)	0 (0.0)	0 (0.0)
Hyperbilirubinaemia	13 (16.3)	9 (11.3)	4 (5.0)	0 (0.0)	0 (0.0)	0 (0.0)
Acute renal toxicity	1 (1.3)	1 (1.3)	0 (0.0)	0 (0.0)	0 (0.0)	0 (0.0)
Deep-vein thrombosis	3 (3.8)	0 (0.0)	3 (3.8)	0 (0.0)	0 (0.0)	0 (0.0)
Allergic reaction	0 (0.0)	0 (0.0)	0 (0.0)	0 (0.0)	0 (0.0)	0 (0.0)
Pancreatitis	0 (0.0)	0 (0.0)	0 (0.0)	0 (0.0)	0 (0.0)	0 (0.0)

## Discussion

The best front-line treatment for ENKTL remains a matter of debate. Our results indicate that COEPL regimen in combination with radiotherapy is highly effective and safe for patients with both early-stage and advanced-stage ENKTL, suggesting this regimen may be a preferable option for the treatment of ENKTL.

A number of non-anthracycline-containing regimens have been evaluated prospectively in patients with ENKTL in the first-line setting. In patients with limited-stage ENKTL, concurrent chemoradiation with DeVIC regimen (dexamethasone, etoposide, ifosfamide and carboplatin) produced a CR rate of 77% and 2-year OS of 78% (12). Concurrent chemoradiation followed by VIPD regimen (etoposide, ifosfamide, cisplatin and dexamethasone) reported a CR rate of 80% and estimated 3-year OS of 86% (13). Concurrent chemoradiation followed by VIDL regimen (etoposide, ifosfamide, dexamethasone and L-asparaginase) yielded a CR rate of 87% and estimated 5-year OS of 73% (14). Similarly, concurrent chemoradiation plus L-asparaginase followed by MIDLE regimen (methotrexate, ifosfamide, dexamethasone, L-asparaginase and etoposide) showed a CR rate of 82.1% and estimated 3-year OS of 81.5% (15). Sandwich chemoradiation with GELOX regimen (L-asparaginase, gemcitabine and oxaplatin) resulted in a CR rate of 74% and 5-year OS of 85% (16). In patients with advanced-stage ENKTL, the SMILE regimen (dexamethasone, methotrexate, ifosfamide, L-asparginase and etoposide) produced a CR rate of 45% and 1-year OS of 55% (8). The AspaMetDex regimen (L-asparginase, methotrexate and dexamethasone) resulted in a CR rate of 61% and 1-year OS of 50% (17). The P-GEMOX (pegaspargase, gemcitabine and oxaliplatin) plus thalidomide regimen resulted in a CR rate of 56% and 3-year OS of 44.3% (18). Other non-anthracycline-containing regimens reported in the literature yielded similar survival outcomes, with OS ranging from 70-90% for patients with early-stage disease and 40-50% for patients with advanced-stage disease (19–22).

In the present study, the COEPL regimen resulted in CR rates of 82.0% and 55.6%, and 3-year OS of 81.2% and 45.7% for patients with early-stage and advanced-stage ENKTL, respectively. These data were compatible with outcomes of the other non-anthracyline-based regimens published in the literature. With respect to adverse events, the COEPL regimen presented a favorable toxicity profile. Grade 3 or 4 hematologic adverse events (e.g. anemia, neutropenia and thrombocytopenia) were relatively infrequent, occurring in generally less than 30% of patients in our study. These results compare favorably with those reported for gemcitabine-based regimens and are considerably better than those reported for the SMILE or MetAspDex regimens (8, 17, 18). The disparity in toxicity profile might be partially attributable to the differences in the baseline patient characteristics between these studies. For instance, our study has a lower proportion of patients with advanced age (>60 years) and less patients with advanced-stage disease compared with the studies reported for SMILE or MetAspDex regimens (8, 17). In addition, our study enrolled only treatment-naive patients whereas the studies of SMILE or MetAspDex regimens included a proportion of patients with relapsed/refractory disease (8, 17). Nevertheless, our data indicate that the COEPL regimen can be well-tolerated by medically-fit patients with newly diagnosed ENKTL.

A small group of patients presented with primary extra-nasal disease in our study, and treatment outcomes for these patients appear to be favorable compared with historical data (2). Of the 6 patients with primary extra-nasal disease, 4 patients achieved CR after first-line treatment and remained disease-free after follow-up duration of 20-38 months. Historically, primary extra-nasal ENKTL was associated with much worse survival compared with nasal cases when treated with anthracyline-based chemotherapy (5-year OS 9% vs 42%) (2). However, treatment outcomes of extra-nasal ENKTL in the era of non-anthracycline-based chemotherapy remains largely unclear. Although limited by small sample size, our findings raise the question of whether outcome of primary extra-nasal ENKTL might be improved by pegaspargase-based non-anthracyline-containing regimens. This hypothesis would, of course, require validation by future studies based on larger sample size.

Prognostic analyses showed that the PINK score was significantly associated with CR rate, PFS and OS in our cohort. PINK was developed *via* an international multicenter study as a prognostic model for patients with newly diagnosed ENKTL treated with non-anthracycline-based chemotherapy (23). Our data confirmed that PINK score was predictive of both short-term treatment response and long-term survival. Despite excellent survival of patients in the low-risk PINK group, patients in the intermediate- or high-risk group had significantly inferior 3-year PFS and OS. These findings underscore the need for optimization of therapeutic strategies for high-risk ENKTL patients. One possible approach is the employment of consolidative ASCT for high-risk patients who are responsive to induction chemotherapy. In our analyses, of the 6 patients with intermediate/high-risk PINK score who underwent ASCT at remission, 5 patients were disease-free at follow-up of 20-57 months. These results were in line with published data showing greater survival benefit with transplant for patients with high-risk ENKTL (24). An alternative solution is the combination of chemotherapy with novel targeted agents such as PD-1 antibodies, histone deacetylase inhibitors or CD30-antibody conjugates (25–27). In a phase 2 trial, PD-1 antibody monotherapy produced an ORR of 68% and 1-year OS of 82% in patients with relapsed/refractory ENKTL (25). Other clinical trials have reported promising early results of the combination of a PD-1 antibody with a histone deacytelase inhibitor for relapsed/refractory ENKTL (27). Although these agents have not yet been tested in the first-line setting, it is hopeful that results from these trials might eventually change the treatment landscape of ENKTL towards a more risk-adapted and individualized approach.

This study has several limitations. Firstly, this is a single-arm study which consisted of mainly patients with low- or intermediate-risk ENKTL, thus results from this study cannot be directly compared with the other regimens reported in the literature. Secondly, EBV-DNA testing was not routinely performed in this study, thus the impact of EBV-DNA level on risk stratification and treatment outcome cannot be analyzed. Thirdly, the group of patients with advanced-stage disease was relatively small (N=16), thus it is difficult to adequately evaluate the efficacy of COEPL regimen in this group of patients. In light of this limitation, we have initiated another prospective study of COEPL chemotherapy in patients with newly diagnosed stage III/IV ENKTL, and results of this ongoing trial will be presented as a separate report.

In conclusion, our study demonstrated that COEPL chemotherapy is highly effective and well-tolerated for patients with newly diagnosed ENKTL. This regimen offers a safe and effective alternative treatment option for the front-line treatment of ENKTL.

## Data Availability Statement

The raw data supporting the conclusions of this article will be made available by the authors, without undue reservation.

## Ethics Statement

The studies involving human participants were reviewed and approved by the Ethical Review Committee of Peking University Cancer Hospital. Written informed consent to participate in this study was provided by the participants’ legal guardian/next of kin.

## Author Contributions

SH, NL, JZ, and YQS contributed to the conception and design. All authors contributed to the acquisition of data. SH and NL contributed to the statistical analysis and interpretation of data. All authors contributed in drafting the article or revising it critically for important intellectual content. All authors gave the final approval of the version published.

## Funding

This study was funded by Beijing Municipal Health Commission No.2018KT84.

## Conflict of Interest

Author YW was employed by Medpison (Beijing) Medical Technology Co., Ltd.

The remaining authors declare that the research was conducted in the absence of any commercial or financial relationships that could be construed as a potential conflict of interest.

## Publisher’s Note

All claims expressed in this article are solely those of the authors and do not necessarily represent those of their affiliated organizations, or those of the publisher, the editors and the reviewers. Any product that may be evaluated in this article, or claim that may be made by its manufacturer, is not guaranteed or endorsed by the publisher.

## Abbreviations

ENKTL, extra-nodal NK/T-cell lymphoma; CR, complete response; PR, partial response; SD, stable disease; PD, progressive disease; ORR, objective response rate; PFS, progression-free survival; OS, overall survival; ASCT, autologous stem cell transplant; PINK, Prognostic Index for Natural Killer Lymphoma; CT, computed tomography; PET, positron emission tomography; MRI, magnetic resonance imaging.

## References

[1] Vose J Armitage J Weisenburger D. International Peripheral T-Cell and Natural Killer/T-Cell Lymphoma Study: Pathology Findings and Clinical Outcomes. J Clin Oncol (2008) 26:4124–30. doi: 10.1200/JCO.2008.16.4558 18626005

[2] Au WY Weisenburger DD Intragumtornchai T Nakamura S Kim WS Sng I. Clinical Differences Between Nasal and Extranasal Natural Killer/T-Cell Lymphoma: A Study of 136 Cases From the International Peripheral T-Cell Lymphoma Project. Blood (2009) 113(17):3931–7. doi: 10.1182/blood-2008-10-185256 19029440

[3] Tse E Kwong YL. The Diagnosis and Management of NK/T-Cell Lymphomas. J Hematol Oncol (2017) 10:85. doi: 10.1186/s13045-017-0452-9 28410601 PMC5391564

[4] Li XY Yao B Jin J Wang WH Liu YP Song YW. Radiotherapy as Primary Treatment for Stage IE and IIE Nasal Natural Killer/T-Cell Lymphoma. J Clin Oncol (2006) 24:181–9. doi: 10.1200/JCO.2005.03.2573 16382127

[5] Yang Y Zhu Y Cao JZ Zhang YJ Xu LM Yuan ZY. Risk-Adapted Therapy for Early-Stage Extranodal Nasal-Type NK/T-Cell Lymphoma: Analysis From a Multicenter Study. Blood (2015) 126:1424–32. doi: 10.1182/blood-2015-04-639336 26109206

[6] Yamaguchi M Suzuki R Oguchi M. Advances in the Treatment of Extra-Nodal NK/T-Cell Lymphoma, Nasal Type. Blood (2018) 131:2528–40. doi: 10.1182/blood-2017-12-791418 29602763

[7] Yamaguchi M Kita K Miwa H Nishii K Oka K Ohno T. Frequent Expression of P-Glycoprotein/MDR1 by Nasal T-Cell Lymphoma Cells. Cancer (1995) 76(11):2351–6. doi: 10.1002/1097-0142(19951201)76:11<2351::AID-CNCR2820761125>3.0.CO;2-1 8635042

[8] Yamaguchi M Kwong YL Kim WS Maeda Y Hashimoto C Suh C. Phase II Study of SMILE Chemotherapy for Newly Diagnosed Stage IV, Relapsed, or Refractory Extranodal Natural Killer (NK)/T-Cell Lymphoma, Nasal Type: The NK-Cell Tumor Study Group Study. J Clin Oncol (2011) 29(33):4410–6. doi: 10.1200/JCO.2011.35.6287 21990393

[9] Lin N Song Y Zheng W Tu M Xie Y Wang X. A Prospective Phase II Study of L-Asparaginase-CHOP Plus Radiation in Newly Diagnosed Extranodal NK/T-Cell Lymphoma, Nasal Type. J Hematol Oncol (2013) 6:44. doi: 10.1186/1756-8722-6-44 23816178 PMC3734195

[10] Zheng W Gao Y Ke X Zhang W Su L Ren H. PEG-L-CHOP Treatment is Safe and Effective in Adult Extranodal NK/T-Cell Lymphoma With a Low Rate of Clinical Hypersensitivity. BMC Cancer (2018) 18(1):910. doi: 10.1186/s12885-018-4782-y 30241515 PMC6151061

[11] Cheson BD Fisher RI Barrington SF Cavalli F Schwartz LH Zucca E. Recommendations for Initial Evaluation, Staging, and Response Assessment of Hodgkin and Non-Hodgkin Lymphoma: The Lugano Classification. J Clin Oncol (2014) 32(27):3059–68. doi: 10.1200/JCO.2013.54.8800 PMC4979083 25113753

[12] Yamaguchi M Tobinai K Oguchi M Ishizuka N Kobayashi Y Isobe Y. Phase I/II Study of Concurrent Chemoradiotherapy for Localized Nasal Natural Killer/T-Cell Lymphoma: Japan Clinical Oncology Group Study Jcog0211. J Clin Oncol (2009) 27(33):5594–600. doi: 10.1200/JCO.2009.23.8295 19805668

[13] Kim SJ Kim K Kim BS Kim CY Suh C Huh J. Phase II Trial of Concurrent Radiation and Weekly Cisplatin Followed by VIPD Chemotherapy in Newly Diagnosed, Stage IE to IIE, Nasal, Extranodal NK/T-Cell Lymphoma: Consortium for Improving Survival of Lymphoma Study. J Clin Oncol (2009) 27(35):6027–32. doi: 10.1200/JCO.2009.23.8592 19884539

[14] Kim SJ Yang DH Kim JS Kwak JY Eom HS Hong DS. Concurrent Chemoradiotherapy Followed by L-Asparaginase-Containing Chemotherapy, VIDL, for Localized Nasal Extranodal NK/T Cell Lymphoma: CISL08-01 Phase II Study. Ann Hematol (2014) 93(11):1895–901. doi: 10.1007/s00277-014-2137-6 24947798

[15] Yoon DH Kim SJ Jeong SH Shin DY Bae SH Hong J. Phase II Trial of Concurrent Chemoradiotherapy With L-Asparaginase and MIDLE Chemotherapy for Newly Diagnosed Stage I/II Extranodal NK/T-Cell Lymphoma, Nasal Type (CISL-1008). Oncotarget (2016) 7(51):85584–91. doi: 10.18632/oncotarget.11319 PMC5356760 27542213

[16] Wang L Wang ZH Chen XQ Wang KF Huang HQ Xia ZJ. First-Line Combination of GELOX Followed by Radiation Therapy for Patients With Stage IE/IIE ENKTL: An Updated Analysis With Long-Term Follow-Up. Oncol Lett (2015) 10(2):1036–40. doi: 10.3892/ol.2015.3327 PMC4509369 26622621

[17] Jaccard A Gachard N Marin B Rogez S Audrain M Suarez F. Efficacy of L-Asparaginase With Methotrexate and Dexamethasone (AspaMetDex Regimen) in Patients With Refractory or Relapsing Extranodal NK/T-Cell Lymphoma, a Phase 2 Study. Blood (2011) 117(6):1834–9. doi: 10.1182/blood-2010-09-307454 21123825

[18] Huang H Gao Y Su H Huang Y Gao Y Wang X. Clinical Outcome of a Prospective, Multicenter, Randomized, Phase III Non-Inferiority Clinical Trial for Patients With Extranodal NK/T Cell Lymphoma Treated by P-GEMOX or AspaMetDex. Hematol Oncol (2019) 37(S2):161–3. doi: 10.1002/hon.119_2629

[19] Wang JH Wang L Liu CC Xia ZJ Huang HQ Lin TY. Efficacy of Combined Gemcitabine, Oxaliplatin and Pegaspargase (P-Gemox Regimen) in Patients With Newly Diagnosed Advanced-Stage or Relapsed/Refractory Extranodal NK/T-Cell Lymphoma. Oncotarget (2016) 7(20):29092–101. doi: 10.18632/oncotarget.8647 PMC5045380 27093153

[20] Kwong YL Kim SJ Tse E Oh SY Kwak JY Eom HS. Sequential Chemotherapy/Radiotherapy was Comparable With Concurrent Chemoradiotherapy for Stage I/II Nk/T-Cell Lymphoma. Ann Oncol (2018) 29:256–63. doi: 10.1093/annonc/mdx684 29077846

[21] Wang L Wang WD Xia ZJ Xia ZJ Zhang YJ Xiang J. Combination of Gemcitabine, L-Asparaginase, and Oxaliplatin (GELOX) is Superior to EPOCH or CHOP in the Treatment of Patients With Stage IE/IIE Extranodal Natural Killer/T Cell Lymphoma: A Retrospective Study in a Cohort of 227 Patients With Long-Term Follow-Up. Med Oncol (2014) 31:860. doi: 10.1007/s12032-014-0860-4 24481637

[22] Zhang L Jia S Ma Y Li L Li X Wang X. Efficacy and Safety of Cisplatin, Dexamethasone, Gemcitabine and Pegaspargase (DDGP) Regimen in Newly Diagnosed, Advanced-Stage Extranodal Natural Killer/T-Cell Lymphoma: Interim Analysis of a Phase 4 Study NCT01501149. Oncotarget (2016) 7(34):55721–31. doi: 10.18632/oncotarget.10124 PMC5342448 27384676

[23] Kim SJ Yoon DH Jaccard A Chng WJ Lim ST Hong H. A Prognostic Index for Natural Killer Cell Lymphoma After Non-Anthracycline-Based Treatment: A Multicentre, Retrospective Analysis. Lancet Oncol (2016) 17(3):389–400. doi: 10.1016/S1470-2045(15)00533-1 26873565

[24] Lee J Au WY Park MJ Suzumiya J Nakamura S Kameoka J. Autologous Hematopoietic Stem Cell Transplantation in Extranodal Natural Killer/T Cell Lymphoma: A Multinational, Multicenter, Matched Controlled Study. Biol Blood Marrow Transplant (2008) 14(12):1356–64. doi: 10.1016/j.bbmt.2008.09.014 19041057

[25] Tao R Fan L Song Y Hu Y Zhang W Wang Y. Sintilimab for relapsed/refractory (r/r) Extranodal NK/T-Cell Lymphoma (ENKTL): A Multicenter, Single-Arm, Phase 2 Trial (ORIENT-4). Hematol Oncol (2019) 37(S2):102–3.

[26] Kim HK Moon SM Moon JH Park JE Byeon S Kim WS. Complete Remission in CD30-Positive Refractory Extranodal NK/T-Cell Lymphoma With Brentuximab Vedotin. Blood Res (2015) 50(4):254–6. doi: 10.5045/br.2015.50.4.254 PMC4705052 26770954

[27] Huang H Gao Y Wang X Bai B Zhang L Xiao Y. Sintilimab Plus Chidamide for Relapsed or Refractory(R/R) Extranodal NK/T Cell Lymphoma (ENKTL): A Prospective, Multicenter, Single-Arm, Phase IB/II Trial (SCENT). Hematol Oncol (2021) 39(S2). doi: 10.1002/hon.127_2880

